# Noninvasive Detection of Extracellular pH in Human Benign and Malignant Liver Tumors Using CEST MRI

**DOI:** 10.3389/fonc.2020.578985

**Published:** 2020-11-02

**Authors:** Yanyan Tang, Gang Xiao, Zhiwei Shen, Caiyu Zhuang, Yudan Xie, Xiaolei Zhang, Zhongxian Yang, Jitian Guan, Yuanyu Shen, Yanzi Chen, Lihua Lai, Yuanfeng Chen, Shuo Chen, Zhuozhi Dai, Runrun Wang, Renhua Wu

**Affiliations:** ^1^ Department of Medical Imaging, The Second Affiliated Hospital, Shantou University Medical College, Shantou, China; ^2^ Department of Medical Imaging, The Seventh Affiliated Hospital of Sun Yat-sen University, Shenzhen, China; ^3^ Department of Mathematics and Statistics, Hanshan Normal University, Chaozhou, China; ^4^ Philips Healthcare, Beijing, China; ^5^ Department of General Surgery, The Second Affiliated Hospital, Shantou University Medical College, Shantou, China; ^6^ College of Air Traffic Management, Civil Aviation Flight University of China, Guanghan, China; ^7^ Provincial Key Laboratory of Medical Molecular Imaging, Shantou, China

**Keywords:** extracellular pH, ioversol, chemical exchange saturation transfer magnetic resonance imaging (MRI), hepatic carcinoma, hepatic hemangioma

## Abstract

**Purpose:**

In this study, we aimed to use 3T magnetic resonance imaging (MRI), which is clinically available, to determine the extracellular pH (pHe) of liver tumors and prospectively evaluate the ability of chemical exchange saturation transfer (CEST) MRI to distinguish between benign and malignant liver tumors.

**Methods:**

Different radiofrequency irradiation schemes were assessed for ioversol-based pH measurements at 3T. CEST effects were quantified *in vitro* using the asymmetric magnetization transfer ratio (MTRasym) at 4.3 ppm from the corrected Z spectrum. Generalized ratiometric analysis was conducted by rationing resolved ioversol CEST effects at 4.3 ppm at a flip angle of 60 and 350°. Fifteen patients recently diagnosed with hepatic carcinoma and five patients diagnosed with hepatic hemangioma [1 male; mean age, 48.6 (range, 37–59) years] were assessed.

**Results:**

By conducting dual-power CEST MRI, the pH of solutions was determined to be 6.0–7.2 at 3T *in vitro*. In vivo, ioversol signal intensities in the tumor region showed that the extracellular pH in hepatic carcinoma was acidic(mean ± standard deviation, 6.66 ± 0.19), whereas the extracellular pH was more physiologically neutral in hemangioma (mean ± standard deviation, 7.34 ± 0.09).The lesion size was similar between CEST pH MRI and T2-weighted imaging.

**Conclusion:**

dual-power CEST MRI can detect extracellular pH in human liver tumors and can provide molecular-level diagnostic tools for differentiating benign and malignant liver tumors at 3T.

## Introduction

Solid tumors have been reported to have a slightly acidic extracellular pH (pH_e_) range of 6.4–6.9, while the pH of normal tissues is neutral and ranges from 7.2–7.5 (1, 2). Metabolic dysregulation significantly contributes to glycolytic processes, causing excess intracellular lactic acid accumulation in tumors. Similarly, perfusion of highly angiogenic tumors can also cause lactic acid build-up (3, 4). Solid tumor acidosis enhances tumor aggressiveness and metastasis (5). Studies of preclinical models and clinical cases have shown that the tumor region has an acidic pH_e_ and an alkaline intracellular pH (pH_i_) (6–8). An acidic tumor pH_e_ increases therapeutic resistance, while an alkaline pHi helps maintain resistance to cytotoxicity (9, 10). Further, the weak base chemotherapeutic agent esomeprazole has a strong effect on acidic tumors, while the weak acid chemotherapeutic agent Adriamycin has a poor effect (11, 12). Previous studies on alkalinizing treatments have reported that bicarbonate therapy significantly reduces metastasis in mouse models of breast and prostate cancer, primarily by decreasing the release of active cathepsin B into the pericellular space (13, 14). However, alkalinizing treatments, which are required for tumor pH_e_ normalization, potentially cause metabolic alkalosis at high doses. Therefore, accurate measurement of the pH_e_ of tumors and normal tissue can help regulate alkalinizing treatment.

Despite the fact that the pH_e_ of solid tumors can be measured noninvasively *in vivo via* several imaging methods, improved clinical imaging methods are required. For instance, optical imaging can measure tumor pH_e_ with high sensitivity, but only in the tumor surface (15, 16). Similarly, positron emission tomography (PET), conventional and hyperpolarized magnetic resonance spectroscopy (MRS) (17–19), and electron paramagnetic resonance spectroscopy (EPR) (20) can measure pH, but have limited accuracy and require improved sensitivity and spatial resolution. Novel, noninvasive, precise, and clinically relevant methods are therefore required for improved measurement of tumor pH_e_ *in vivo*.

Chemical exchange saturation transfer (CEST) imaging is a promising strategy for tissue pH quantification. Conventional ratiometric CEST imaging minimizes the confounding concentration factor by analyzing different CEST effects from various exchangeable groups, and requires CEST agents with multiple magnetically non-equivalent protons (21–24). Longo and Sun et al. proposed an improved ratio method based on different saturation intensities. This method does not require a contrast agent to have two CEST signals, but rather changes the intensity of the RF pulse, thus increasing the diversity of contrast agents used in CEST pH-based technologies (25). Moreover, recently developed RF power-based ratiometric methods have been quantitated and optimized (26, 27), facilitating their *in vivo* translation.

Ioversol is a widely used non-ionic X-ray contrast agent with high hydrophilicity and low toxicity and has been previously used in magnetic resonance imaging in a liver cancer model (26, 27). Jones et al. demonstrated that acidoCEST MRI can accurately measure tumor pH, and can be used to clinically evaluate patients with metastatic ovarian cancer (28). However, to the best of our knowledge, there are no reports of *in vivo* studies of CEST pH imaging focusing on the characteristics of liver tumors and the differential diagnosis of benign and malignant liver tumors under 3T low-field strength.

Continuous advancements in various imaging technologies have increased the detection rate of liver tumors (29). Liver cancer is the second most common cause of cancer-related mortality worldwide (30), and the imaging phenotypes of some liver cancers are similar to other liver tumors, thus leading to misdiagnosis. Therefore, it is very important to distinguish between malignant and benign liver lesions. As a new molecular imaging modality, dual-power CEST MRI potentially provides information regarding the tumor microenvironment.

Previous research suggests that 7T chemical exchange saturation transfer (CEST) magnetic resonance imaging (MRI) using ioversol can measure the extracellular pH (pHe) in liver cancer and breast cancer models with good spatial resolution (26, 27). In this study, we aimed to translate ioversol CEST pH MRI from high- to low-field strengths. To investigate its clinical utility, we performed a radiofrequency (RF) power-based ratiometric CEST MRI (dual-power CEST MRI), using a 3T MRI scanner for patients with either hepatic carcinoma or hepatic hemangioma. We hypothesized that CEST pH imaging may be a potential diagnostic tool for differential diagnosis of hepatic carcinoma and hepatic hemangioma. Therefore, the purpose of this study was to prospectively evaluate the ability of CEST pH imaging to characterize liver tumor lesions in a comparative study.

## Materials and Methods

### Phantom Preparation

To assess whether CEST pH imaging is affected by the concentration of ioversol, ioversol (Hengrui Medicine Co., Ltd., Jiangsu, China) was diluted with phosphate-buffered saline (PBS) to concentrations ranging from 30–110 mM in 20 mM units. The pH was adjusted to 7.20 using 6 M HCl and 6 M NaOH. The addition of <0.3 ml HCl and NaOH to 50 ml of each solution slightly influenced the sample concentration. To obtain the measurable pH range for *in vitro* experiments, five additional cylinders containing 50 mM ioversol were dissolved in PBS. The prepared solution was titrated to pH values of 6.0, 6.3, 6.6, 6.9, and 7.2. The phantom was maintained at 37.0 ± 0.2°C.

### Patients

This study was approved by the Committee of the Second Affiliated Hospital of Shantou University Medical College. The human experiments complied with the ethical standards of the Declaration of Helsinki 1964. All patients provided signed informed consent. If patients with limited mobility were unable to provide consent, consent was obtained from their accompanying family members. Any patients with contraindications to MRI testing were excluded from the study. The testing process and study protocol were clearly explained to each participant prior to imaging. From September 2017 to August 2019, 15 patients (13 males, 2 females; mean age, 63 years; age range, 49–75 years) with recently diagnosed and histopathologically confirmed hepatic carcinoma were enrolled in the study. An additional five patients diagnosed with hepatic hemangioma (1 male, 4 females; mean age, 48.6 years; age range, 37–59 years) were included in the prospective study.

### MR Imaging and Scanning

MRI data were obtained using a 3.0-T MRI scanner (Sigma; GE Healthcare, Milwaukee, WI, USA). The signal was received through an 8-channel torso coil, and radiofrequency was transmitted using a body coil. To limit body movement, a sponge pad was used to secure the patient during the examination. Conventional T2-weighted imaging was performed to assess tumor anatomy and location, using the following parameters: turbo spin-echo factor = 54; echo time (TE)/repetition time (TR) = 1,236/70 ms; field-of-view (FOV) = 320×320 mm2; slice thickness = 5 mm. CEST images were acquired using RF pulses for presaturation, followed by a gradient-echo readout. An MT-generated GRE MRI sequence with a Fermi pulse [16–32 ms width and B1 of 0.2–2.78 μT (50–850°)] was used herein. For all *in vitro* experiments, different saturation pulses and flip angles were used per their influence on the CEST effect. Here, to ensure clear contrast CEST images, we used different flip angles of 50–850°(0.2–2.78 μT) to generate multiple saturation powers, thus optimizing the conditions. A total of 50 images were acquired, including 49 frequency offsets (0.25 ppm increment in saturation frequency**)** from 6 to -6 ppm and another S0 image without saturation pulses. The B0 correction method uses the Water Saturation Shift Reference (WASSR) method proposed by Kim et al. (31), which applies a small power and short duration RF pulse to negate the MT and CEST effects. This ensures that the direct saturation (DS) effect is the primary readout. Using this method, the frequency offset information for the free water signal, as well as the non-uniform characteristics of the B0 field, can be obtained. Data acquisition lasted 6 min 31 s for 49 offsets, and 2 min for T2-weighted images.

For *in vivo* experiments, the following optimized acquisition parameters were used: TR (sec)/TE (sec), 67/3.1; one slice; 5 mm slice thickness; 15.63 kHz bandwidth; 10° flip angle; 128 × 128 matrix; and 2.4× 2.4 cm2 FOV; 1.88×1.88 in-plane resolution. Two flip angles of 60°(B1 = 0.2 µT) and 350°(B1 = 1.15 µT) were used for CEST imaging. The Fermi pulse with a width of 28 ms was set as the MT saturation pulse. To shorten the scan time and reduce the amount of offset, we excluded 10 offset points (± 5.25, ± 5.5, ± 5.75, ± 6.0 ppm) and used the remaining 41 offsets (5 to -5 ppm) to produce CEST pH images. Ioversol was administered at 1 ml/s for 60 s *via* a catheter inserted in the arm. To reduce the washout effect for pH measurement, the contrast agent was infused at 0.15 ml/s for 5 min during CEST data acquisition. The total injection volume was 105 ml. The patient was instructed to hold his/her breath, and an automated respiratory gating trigger was applied when the breath was stably held. When the patient breathed normally, a 28 ms pre-saturation pulse was applied. When the patient stably held his/her breath, an 8S scan was initiated. Actual data collection requires an average of one breath-hold for each saturation pulse. The time for each patient to hold their breath is approximately 10 s, with approximately 82 breath-holds per patient; thus, the breath-holding duration during the entire examination was approximately 13 min 30 s, the conventional T2-weighted image scanning duration was 2 min, and the entire duration of the examination was approximately 15 min. To improve the success rate of patient compliance, the examinee was trained to hold their breath during the image acquisition process and maintained a similar breathing depth at certain intervals. Nevertheless, owing to the presence of artifacts or incomplete acquisition, the images of three patients could not be used for analysis and were therefore excluded.

### Data Analysis

All relevant image data obtained from experiments were processed using MATLAB 7 (MathWorks, Natick, MA, USA) (32, 33). Drawing of the Z spectrum primarily depends on the normalized water signal intensity (Isat/I0) and the off-resonance frequency of the saturation pulse (34). For CEST analysis, magnetization transfer asymmetry (MTRasym) was used as the metric. This value is often used to represent the size of the CEST effect, and thus to reflect the concentration of the solute. The calculation formula is: MTRasym (ΔCS) = [I (-ΔCS) -I (ΔCS)]/I0; where I0 refers to the image signal intensity obtained when no pre-saturation pulse is applied, and I (Δcs) refers to the pre-saturation pulse signal strength. Herein, 4.3 ppm was set as the chemical shift position of ioversol in MTRasym, and GraphPad Prism software was used for processing. The statistical significance of the data was analyzed using Student’s t-tests, and a value of P <0.05 was considered significant.

**Figure 1:**
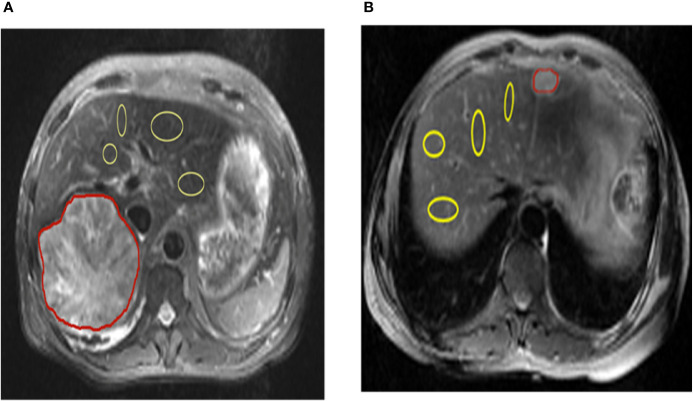
Representation of regions of interest (ROIs). The T2 image shows the position of ROIs in the liver parenchyma in the tumor and tumor-adjacent normal tissue in hepatic carcinoma and hepatic hemangioma, respectively (red lines represent tumor areas, and yellow lines represent non-tumor areas). CEST, chemical exchange saturation transfer.

All patients were assessed independently by three experienced radiologists. The T2-weighted image was used to draw the tumor regions of interest (ROIs) (red marked area) and then apply it to the CEST pH map. In the non-tumor liver parenchymal region of the T2-weighted anatomical image, we selected 4 regions (yellow marked area) of excluded blood vessels and artifacts, each approximately 100–500 mm2, in different ROIs. ROI was copied to the corresponding CEST pH map, and the pH of the area was measured (**Figure 1**).

## Results

### CEST Effects of Ioversol

**Figure 2:**
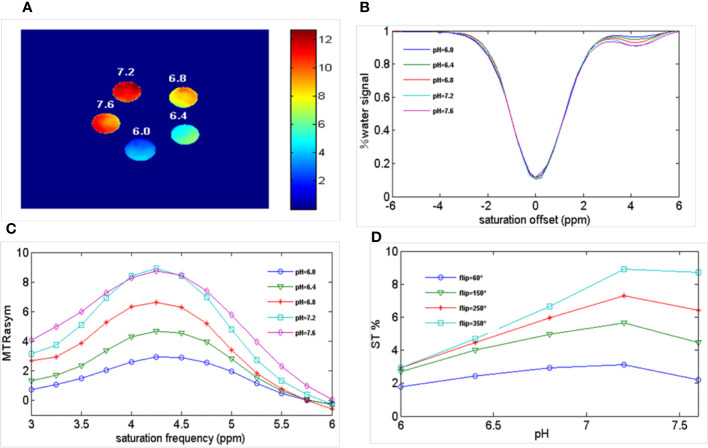
Ioversol exhibits a strong CEST signal. **(A)** CEST map of five different pH phantoms with a flip angle of 350°. **(B)** Z-spectra of 50 mM ioversol at different pH values (6.0, 6.4, 6.8, 7.2, 7.6) with a flip angle of 350°. **(C)** MTR_asym_ curves of 5 different pH ioversol phantoms measured with a flip angle of 350°. **(D)** ST% based on of different pH phantoms with different flip angles. CEST, chemical exchange saturation transfer; MTR_asym_, asymmetric magnetization transfer ratio; ST%, saturation transfer effect.

**Figure 2** **s**hows the results of different pH PBS phantoms. As shown in **Figure 2A**, the CEST effect was pH-dependent and displayed adequate contrast. **Figure 2B** and **Figure 2C** show the Z spectra and MTRasym images of solutions with different pH values. At pH 6.0–7.2, the CEST effect gradually increased with an increase in pH but decreased at pH 7.6, owing to rapid chemical exchange saturation of the solution (35). To evaluate the CEST effect of all different pH solutions at different flip angles, we fitted the transfer effects of solutions of different pH at four flip angles (flip = 60, 150, 250, and 350°; **Figure 2D**). The change in trend was consistent at the current flip angles.

### *In Vitro* Imaging of Ioversol

**Figure 3:**
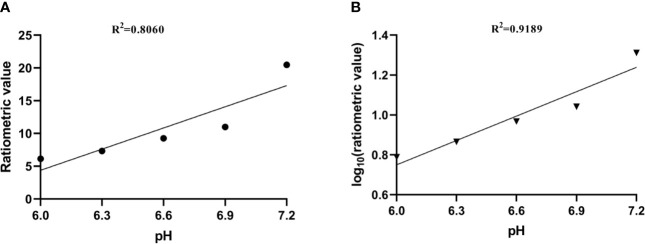
**(A)** CEST ratio was linearly related to pH. **(B)** The log10 ratio of the CEST effect was linearly correlated with the pH. CEST, chemical exchange saturation transfer.

**Figure 4:**
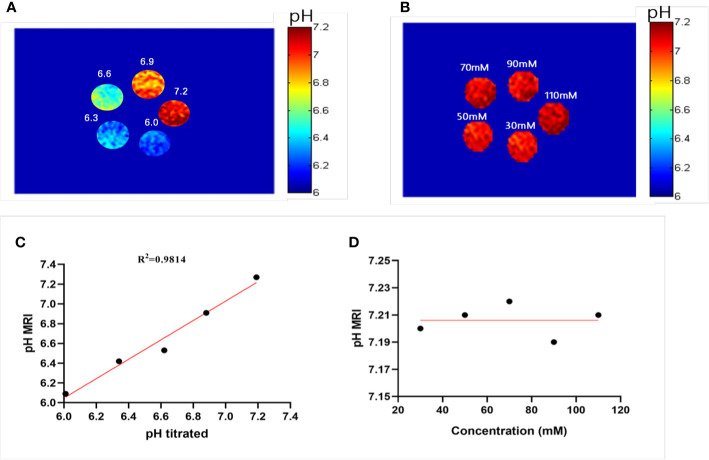
Experimental pH measurements in phantoms. **(A)** The pH map of 50 mM ioversol at pH 6.0, 6.3, 6.6, 6.9, and 7.2, determined using ST images at flip angles of 60 and 350°. **(B)** pH map of ioversol at different concentrations (30, 50, 70, 90, and 110 mM), determined using ST images at flip angles of 60 and 350°. **(C)** The pH values determined herein correlated with the titrated pH values. **(D)** Mean pH values determined for several concentrations. ST, saturation transfer.

We performed ioversol pH MRI for a phantom with pH titrated to 6.0 and 7.2 at two flip angles (60 and 350°). Consequently, the ratiometric value was obtained using Equation 1:1

(1) $$RPM=\frac{{\left[\frac{1-ST}{ST}\right]}_{RF1}}{{\left[\frac{1-ST}{ST}\right]}_{RF2}}$$

where ST_RF1,2_ are the saturation transfers obtained at different flip angles. The saturation transfer (ST) effect is matched at two different flip angles to determine the RF power mismatch. We optimized the number of saturations, TR, TE, and bandwidth by comparing the Z spectrum, MTR_asym_ spectrum, and the CEST imaging of the phantoms. Finally, we used different flip angles with other fixed parameters to achieve different RF irradiation power. In short, the RPM calculated from the RF power levels generated by the flip angles of 60 and 350° can provide relatively good pH sensitivity and range. The RF power mismatch curve revealed that the ratiometric values increased linearly for pH values ranging from 6.0–7.2 (**Figure 3A**). The log_10_ ratio of the CEST effect generated at the RF power corresponding to the 60 and 350° flip angles shows an excellent correlation with the model pH (R^2^ = 0.9189, *P* < 0.001) (**Figure 3B**). The pH map shows that the signals produced by ioversol solutions at different pH values are significantly different (**Figure 4A**). At pH 6.0–7.2, the pH map revealed a suitable association between pH values (**Figure 4C**). Ioversol dual-power CEST MRI is sensitive to changes in pH at 3T, and the measurable pH range is 6.0–7.2. However, we must acknowledge that pH values were measured at a narrow range using 3T compared to the range used for 7T.

A previous study reported that pH quantification *via* ioversol CEST imaging is concentration-independent at 7T (26). To determine its feasibility under lower field strengths, a phantom was prepared using different concentrations of ioversol at 30−110 mM, with the pH titrated to 7.2. Using the proposed methods, CEST signal changes were determined for the ioversol PBS phantom at flip angles of 60 and 350°. We can see from **Figures 4B, D** that the pH map was not significantly different between the phantoms. The pH values of the ioversol PBS phantom were obtained within a small margin of error, even at an almost 4-fold difference in concentration. This is consistent with the results of our 7T *in vitro* experiment, indicating that this method could be translated from high- to low-field strengths (26).

### *In Vivo* Imaging of Liver Tumors

**Figure 5:**
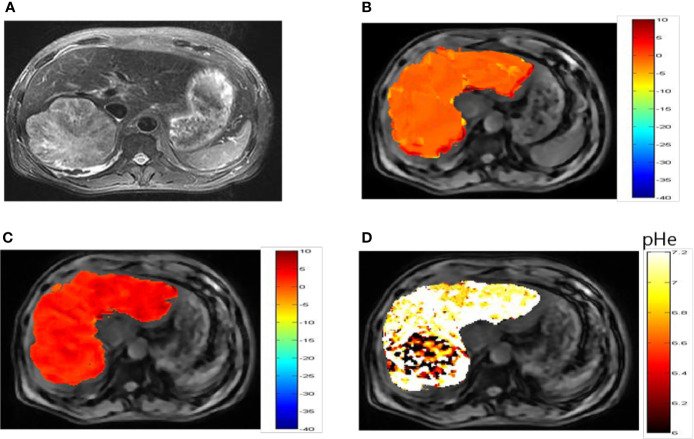
pH_e_ values of hepatic carcinoma using dual-power CEST MRI. **(A)** A representative image of a patient with hepatic carcinoma. **(B, C)** are CEST maps of hepatic carcinoma at flip angles of 60 and 350°. **(D)** pH_e_ map for the tumor volume. The CEST signal for the tumor area differed from the corresponding color of the adjacent normal liver tissue, with a lower pH_e_ value in the tumor area than in normal tissue. CEST, chemical exchange saturation transfer; pHe, extracellular pH; MRI, magnetic resonance imaging.

**Figure 6:**
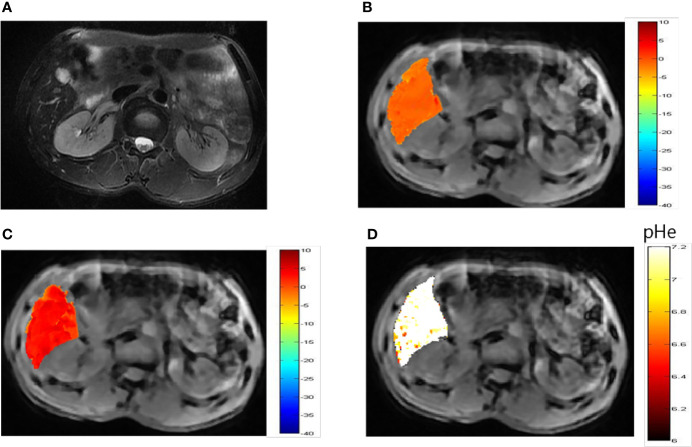
pH_e_ values of hepatic hemangioma using dual-power CEST MRI. **(A)** A representative image of a patient with hepatic hemangioma. CEST image after ioverol injection at flip angles of 60° **(B)** and 350° **(C)**. **(D)** pH_e_ map for hepatic hemangioma; the CEST pH_e_ was consistent with the surrounding liver tissue, confirming the hemangioma to be benign.

Representative images of a hepatocellular carcinoma patient and a hepatic hemangioma patient are presented in **Figures 5A** and **6A**, respectively. **Figures 5B, C** are CEST maps of hepatic carcinoma at flip angles of 60 and 350°. Among the patients with hepatic carcinoma, the CEST signal of the tumor area differed from that of the adjacent liver tissue. This indicates that the pHe value of the tumor area was lower than that of the surrounding normal liver tissue (**Figure 5D**), demonstrating that the present CEST method had a robust performance in the liver tissue. Interestingly, liver tumor area obtained by pH in the CEST pHe image is approximately the same as the area obtained by T2 imaging, which is different from our 7T liver cancer animal model. In the image of hepatic hemangioma, obvious CEST signals were observed at flip angles of 60 and 350° (**Figures 6B, C**, respectively). Among hepatic hemangioma patients, the intensities of the ioversol signals in the CEST pH_e_ analysis were consistent with those in the surrounding liver tissue (**Figure 6D**), suggesting that hepatic hemangioma is a benign tumor with a normal pH_e_ value.

**Figure 7:**
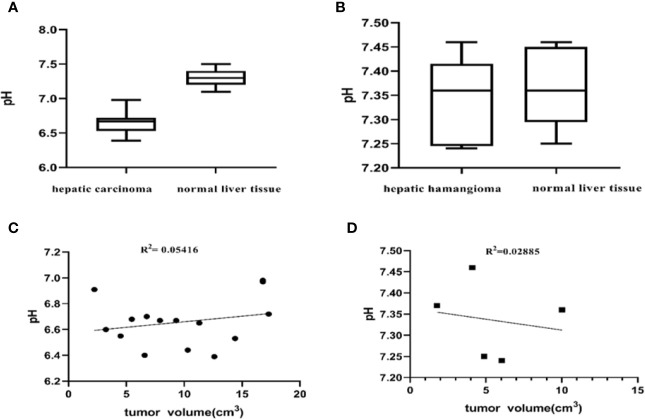
Differences between the tumor region and tumor-adjacent normal liver tissue. **(A)** pH values of the two tissue regions showing a significant difference (*P* < 0.001, Student’s t-test) (n = 15). **(B)** The pH of patients with hepatic hemangioma is almost indistinguishable from that of surrounding normal liver tissue (*P* = 0.5587, Student’s t-test) (n = 5). No significant correlation between the pHe of hepatic carcinoma and hepatic hemangioma and tumor size was observed herein **(C, D)**.

To elucidate differences in ioversol signal intensities in common benign and malignant liver tumors, we evaluated the differences between the tumor region and tumor-adjacent normal liver tissue of 15 patients diagnosed with hepatic carcinoma and 5 patients diagnosed with hepatic hemangioma using a paired Student’s *t*-test. The pH_e_ of the tumor in all 15 patients diagnosed with hepatic carcinoma was lower than that of tumor-adjacent normal liver tissue (*P* < 0.001, **Figure 7A**). The pH_e_ of the tumors in the five patients diagnosed with hepatic hemangioma was nearly indistinguishable from that of the surrounding normal liver tissue (*P* > 0.05, **Figure 7B**). The CEST pH_e_ values of the tumor area and normal liver tissue in patients with hepatic carcinoma and hepatic hemangioma are compared in **Table 1**. Irrespective of the tumor type, hepatic carcinoma or hepatic hemangioma, no marked correlation was observed between tumor size and pHe (**Figures 7C, D**).

**Table 1:** Comparison of CEST pH_e_ values between tumor area and normal liver tissue in patients with hepatic carcinoma and hepatic hemangioma (mean ± SD).

Tumor types	n	Tumor area	Normal liver tissue	*P* value
Hepatic carcinoma	15	6.66 ± 0.19	7.31 ± 0.12	*P* < 0.0001
Hepatic hemangioma	5	7.34 ± 0.09	7.37 ± 0.08	*P* = 0.5587

## Discussion

This study shows that dual-power CEST MRI using ioversol is clinically translatable and can help determine the tumor pHe among patients with liver tumors. To our knowledge, this study is the first to report a dual-power CEST MRI method for measuring the pHe in patients with hepatocellular carcinoma. Among all patients with hepatic carcinoma, ioverisol was used to detect lower pH in the carcinoma relative to that in normal tissues. The volume of hepatic carcinoma and hepatic hemangioma did not display a marked correlation with the pHe determined through the present dual-power CEST MRI method. The present results are consistent with those of dual-peak acidoCEST MRI used for animal models (36), probably because the tumors have reached their respective sizes in a smaller volume. The pHe of these tumors remains relatively constant with tumor growth. In the future, by applying the clinical magnetic resonance field strength of ioversol CEST pH imaging, we will evaluate the efficacy of radiofrequency ablation or chemotherapy for liver cancer.

Clinical the dual-peak CEST MRI protocols have been previously applied to determine the pH_e_ in the bladder (37). However, the pHe can be determined with a 3T scanner with iopamidol for pH at 7.0 or below, and with less precision above 7.0. Other studies have reported that clinical the dual-peak acidoCEST MRI using iopamidol with multiple magnetically non-equivalent proton pools can measure the pH_e_ in other organs (28). A previous study has proposed a Bloch fitting method, which measures a pH range of 6.2–7.0 *in vitro* and *in vivo*. Since the two CEST signals generated by ioversol come from differing and non-conflicting RF levels, our method does not require two different CEST signals in the same CEST spectrum. This further confirms the applicability of our proposed method at lower field strengths. In addition, previous reports suggest that the higher CEST effects of ioversol are due to its faster chemical exchange rate and higher accumulation than iodixanol and iomeprol, allowing clinical translation of ioversol dual-power CEST MRI (35).

Amide proton transfer (APT) has been developed from CEST imaging and has been described by many researchers. APT-CEST-MRI is highly sensitive to pH changes and can clinically evaluate ischemic stroke; hence, APT is associated with a lower pHe because CEST MRI soon after an ischemic events does not alter the mobile protein concentration in the lesion relative to the surrounding tissue (38, 39). It is also used to monitor tumor pH (40). However, due to the opposing influence of tumor acidosis and the mobile protein content of tumors, endogenous CEST MRI of some solid tumors shows no significant difference in CEST contrast between the tumor area and surrounding normal tissues (41). APT MRI is more likely to be negatively affected by other conditions such as endogenous T1 relaxation time of tissues and saturation power, thus yielding inaccurate pHe measurements (40). The mobile amine protons in proteins and peptides can generate CEST signals ranging 2.75–3.0 ppm. Chemical exchange of amide protons and amine protons with water is base-catalyzed, generating robust CEST signals at a low pH. Therefore, some researchers use the ratio method for CEST signals of amide and amine protons [called amine and amide concentration-independent detection (AACID) method] to determine the intratumoral pH (42). Although the CEST signal of the amide proton at 3.50 ppm can be observed using a 3T MRI scanner, it is difficult to detect at 2.75 ppm. The signal at 2.75 ppm is also susceptible to the DS signal of the affected water proton (43). Therefore, clinical imaging of endogenous CEST contrast agents remains challenging (44, 45).

Our study has several limitations. First, although dual-power CEST MRI has potential efficacy for quantitatively diagnosing liver tumors, we did not analyze in detail the reasons that T2 imaging and dual-power CEST MRI of tumor areas are inconsistent with that in the 7T animal model. *In vivo* determination of pH-related molecular biomarkers is needed to further understand the relationship between tumor acidosis and tumor molecular drivers. Second, the ability of dual-power CEST MRI to identify benign and malignant lesions was not directly and prospectively compared with that of other techniques, such as dynamic enhanced CT, fluorodeoxyglucose PET, or PET/CT. Therefore, a direct comparative study of the application of dual-power CEST MRI and other imaging methods should be performed in the future to determine the clinical application value of CEST pH imaging. Third, the study used a small population and evaluated a low number of liver tumor lesions, which affected the statistical results. Further studies with a larger prospective cohort would more accurately determine the diagnostic performance of dual-power CEST MRI.

Moreover, 3 patients were excluded because the CESTpH images were not in the same plane, and the post-processing image signal-to-noise ratio was relatively poor, thus rendering the tumor and adjacent normal tissues decomposed and blurred. Therefore, several limitations of the dual-power approach for CEST MRI measurement of pH should be acknowledged (relative to the dual-peak approach). First, the B1 saturation power throughout the tissue should be exactly the same as the B1 power used for calibration with phantoms. Herein, B1 homogeneity was not evaluated among the phantoms or patients, being a major study limitation. Second, the scan time of the dual-power approach was 2-fold that of the dual-peak approach, thus deterring it clinical translation. Furthermore, the need for two scans raises concerns regarding motion artifacts. Third, the CEST amplitude is low with low-power saturation. Low CEST signals are susceptible to more imprecision, when the CEST contrast approaches the noise level (e.g., low SNR). Therefore, the precision of the dual-power method may be lower than that of the dual-peak method. However, although the present *in vitro* test tube test determined a pH range of 6.0–7.2, we must acknowledge that we have yet to take any important step of evaluating the reliability of our range. Further studies are required to assess the reliability of this *in vivo* pH measurement method. Our MTRasym analysis method is inherently sensitive to the MT or NOE effects in the negative ppm range. Fortunately, the previous research of our team and the animal model studied by Jin et al. showed that NOE is not sensitive to pH (46, 47). Nonetheless, we intend to develop more complex post-processing analytical methods and more advanced scanning methods, thus increasing the reliability of the pHe range measured in our future studies.

In conclusion, CEST pH imaging is a very promising clinical imaging tool that can detect liver tumors based on pH signal information. In addition, the pH value of the liver tumor area and surrounding normal liver tissue can show a significant difference, so it is possible to distinguish hepatic carcinoma from hepatic hemangioma.

## Data Availability Statement

All datasets presented in this study are included in the article/supplementary material.

## Ethics Statement

The studies involving human participants were reviewed and approved by the ethics committee of the Second Affiliated Hospital of Shantou University Medical College. The patients/participants provided their written informed consent to participate in this study.

## Author Contributions

RWu, CZ, and YT designed the study. YT, LL, YaC, and ZY performed the research. ZS, XZ, GX, and YT performed the CEST data analysis. RWu and ZS critically revised the manuscript. All authors contributed to the article and approved the submitted version.

## Funding

This work was supported by the Key Disciplinary Project of Clinical Medicine under the Guangdong High-Level University Development Program (002-18120302). This work was supported by the National Natural Science Foundation of China (Grant No. 31870981) and the Grant for Key Disciplinary Project of Clinical Medicine under the Guangdong High-Level University Development Program (Grant No. 002-18119101). This work was supported by the National Natural Science Foundation of Guangdong (Grant No. 2017A030307020, Grant No. 2017A030313718 and Grant No. 2018A030307057).

## Conflict of Interest

Author ZS was employed by the company Philips Healthcare.

The remaining authors declare that the research was conducted in the absence of any commercial or financial relationships that could be construed as a potential conflict of interest.

## Abbreviations

CEST, chemical exchange saturation transfer; MTR_asym_, asymmetric magnetization transfer ratio; CT, computed tomography; pH_e_, extracellular pH; pH_i_, intracellular pH; PET: positron emission tomography; MRS, magnetic resonance spectroscopy; EPR, electron paramagnetic resonance; RF, radiofrequency; SNR, signal-to-noise ratio; ST%, saturation transfer efficiency; DC, duty cycle
